# Multiple Exciton Generation in Colloidal Nanocrystals

**DOI:** 10.3390/nano4010019

**Published:** 2013-12-24

**Authors:** Charles Smith, David Binks

**Affiliations:** School of Physics and Astronomy and Photon Science Institute, University of Manchester, Manchester M13 9PL, UK; E-Mail: charles.smith-2@manchester.ac.uk

**Keywords:** multiple exciton generation, carrier multiplication, nanocrystals, quantum dots, nanoparticles, solar cells, photovoltaic

## Abstract

In a conventional solar cell, the energy of an absorbed photon in excess of the band gap is rapidly lost as heat, and this is one of the main reasons that the theoretical efficiency is limited to ~33%. However, an alternative process, multiple exciton generation (MEG), can occur in colloidal quantum dots. Here, some or all of the excess energy is instead used to promote one or more additional electrons to the conduction band, potentially increasing the photocurrent of a solar cell and thereby its output efficiency. This review will describe the development of this field over the decade since the first experimental demonstration of multiple exciton generation, including the controversies over experimental artefacts, comparison with similar effects in bulk materials, and the underlying mechanisms. We will also describe the current state-of-the-art and outline promising directions for further development.

## 1. Introduction

In a conventional solar cell much of the energy absorbed from the sun is lost as heat within the first few picoseconds. The solar spectrum is very broad, stretching from the ultraviolet to the infra-red, and so on absorption in a semiconductor most solar photons produce “hot” carriers with energies significantly in excess of the band edge. In bulk semiconductors this excess energy is lost within the first few picoseconds as the carriers cool to the band edge, largely by phonon scattering and emission, becoming waste heat [1]. Carrier cooling is responsible for most of the energy losses in a conventional cell; for instance, in an ideal single-junction cell made from bulk silicon 47% of the incident energy is lost in this way [2]. One strategy to significantly improve the efficiency of a solar cell is to seek ways in which this initial energy loss can be prevented or reduced, and the energy that would otherwise be wasted as heat instead used to increase the output of the cell.

**Figure 1:**
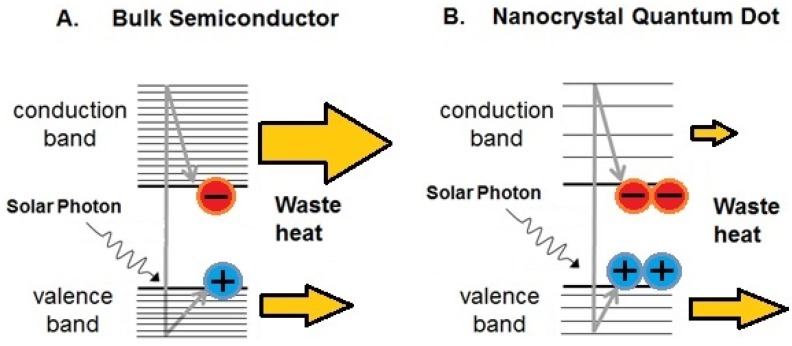
Photogeneration of charges in a (**A**) bulk semiconductor and (**B**) a nanocrystal quantum dot. In (**B**) quantum confinement effects increase the separation between energy levels, inhibiting phonon cooling, enhancing the multiple exciton generation (MEG) process and reducing waste heat.

Impact ionization is a process by which all or some of the excess energy of the hot carriers goes to generate additional electron-hole pairs. (Note that this requires the excess energy to be at least equal to the band gap, *E*_g_). These extra carriers can be extracted from the cell to enhance the photocurrent and thus the overall photovoltaic efficiency. However, it has been long established that impact ionization in bulk semiconductors only becomes a significant process for photon energies much greater than *E*_g_ [3,4,5], and thus does not appreciably improve the efficiency of conventional solar cells. The interest in impact ionization has been revived in recent years with the introduction of colloidal nanocrystals (NCs), semiconductor crystals of sub-micron size. The nano-scale dimensions of NCs can confine the wavefunctions of free electrons and holes such that the conduction and valance bands become discretized into distinct energy levels, in which case they are termed quantum dots (QDs). Moreover, the separation between energy levels increases as the QD size decreases and can become greater than the phonon energy; in this case, cooling by phonon emission must proceed by a much less likely multi-phonon process. It was thus predicted that as the rate of phonon-mediated cooling reduced with decreasing QD size then it would become less dominant and consequently the efficiency of competing processes, such as impact ionization, would improve [6]. (However, later work suggests that at the high energies relevant to multiple exciton generation (MEG) confinement-induced discretization is not important [7], and hence it is not the basis for slowed cooling. The current theoretical understanding of why MEG is enhanced in NCs is discussed in Section 4). Figure 1 compares the photogeneration of charge carriers in a bulk semiconductor and in colloidal NCs.

The first demonstration of impact ionization in QDs, which became known as multiple exciton generation (MEG) or carrier multiplication (CM), was reported in 2004 [8]. A significant number of further studies of MEG, both experimental and theoretical, have been undertaken in the decade since and these are the subject of this review. In particular, the experimental techniques used to measure MEG will be discussed, including the artefacts that confused early results; the results of experimental studies of the dependence of MEG on QD material composition, structure and surface quality will be detailed; and the phenomenological and theoretical descriptions used to understand MEG will also be described. Finally, the direction of future research will be discussed.

## 2. Measuring MEG

MEG can only occur if the photon energy is above a threshold value, *hv*_th_. Above this energy at least one of the charge carriers created by the absorption of a photon has sufficient energy to promote another electron across the band gap, creating a second exciton. The efficiency of this process determines the quantum yield (QY) at a particular photon energy, defined as the average number of excitons created in a QD per photon absorbed; QY > 100% indicates the onset of MEG. MEG is characterized by measuring QY as a function of photon energy normalized to *E*_g_; this relationship is usually well-described by a threshold, *hv*_th_, and a linear rise of gradient η above this threshold.

### 2.1. Ultrafast Spectroscopy

The first challenge in measuring the QY lies in the timescale of the MEG process. Whilst single excitons in QDs have lifetimes of the order 10–100 ns, a multi-exciton can undergo rapid Auger recombination and consequently has a lifetime of the order 10–100 ps. (The Auger recombination process is limited to species containing more than two charge carriers because an additional charge carrier is required to accept the energy liberated from recombination.) QY must thus be measured before the MEG-created excitons can undergo this rapid recombination, necessitating the use of ultrafast techniques. The second challenge lies in the requirement to use low excitation fluences. Multi-excitons can also form when a single QD is successively excited by two or more photons within the lifetime of a single exciton. In order to prevent multi-excitons forming in this way, very low excitation fluences must be used such that the average number of photons absorbed per QD per excitation pulse is much less than unity. The signature of MEG is thus the observation of multi-exciton recombination at low pump fluence. This appears as a sub-nanosecond decay in signal down to a constant level, corresponding to the single-exciton states that remain after multi-exciton recombination is complete and which do not recombine significantly on this timescale. Sensitive detection techniques are consequently required to detect the weak signals resulting from such low excitation. Fortunately, there are a number of spectroscopic techniques available which combine the required sensitivity and ultrafast time-resolution.

#### 2.1.1. Transient Absorption

**Figure 2:**
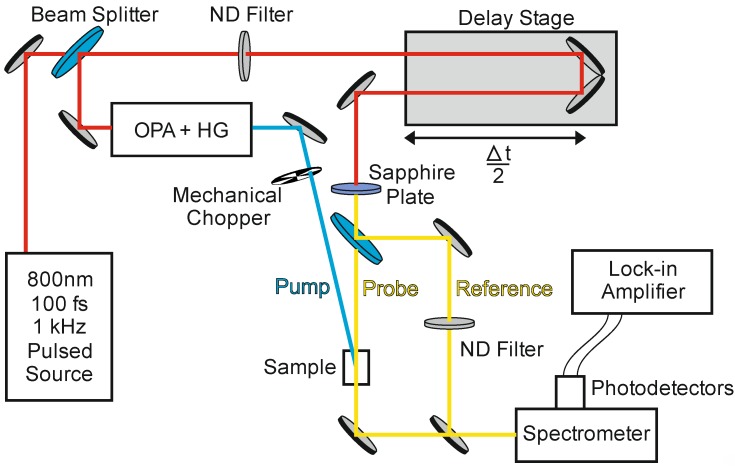
Schematic of a typical ultrafast transient absorption (UTA) experiment for measuring MEG. A Ti:Sapphire regenerative amplifier seeded by a Ti:Sapphire oscillator produces a pulsed beam at 800 nm, with 100 fs pulse width, ~1 mJ pulse energy and 1 kHz repetition rate (red line). Using a beam splitter 95% of this beam is passed to an optical parametric amplifier (OPA) with harmonic generating (HG) crystals to produce a pump beam that is tunable from the infra-red to the ultra-violet (blue line). The pump beam is passed through a mechanical chopper to improve the signal-to-noise ratio and then focused onto the sample. The remaining 5% of the beam from the amplifier is directed through a delay stage to vary the arrival time difference between pump and probe pulses at the sample. This beam is then passed through a sapphire plate to produce a white light continuum, which is split to form the probe and reference beams (yellow line). After the sample, the probe and reference beams are balanced using a neutral density (ND) filter (in the absence of the pump), passed through a spectrometer, then onto separate photodetectors. Small differences in probe and reference are detected using a lock-in amplifier synchronised to the chopper.

Ultrafast transient absorption (UTA) is a pump-probe technique which was first used to measure MEG in 2004 [8]. Sub-picosecond, low fluence pulses of high energy pump photons create initially hot single exciton states. The subsequent population of band-edge excitons, produced either by the dissipative cooling of the hot excitons or by MEG, is then monitored by measuring the absorption of a probe beam tuned to specific electronic transitions within the QD. Band-edge excitons fill the available states, resulting in either an absorption bleach or a photo-induced increase in absorption depending on the transition probed. Varying the delay between the arrival of the pump and probe pulses enables the growth and decay of this band-edge exciton population to be measured. A typical set-up for UTA is shown in Figure 2.

Several variants of UTA have been developed based on the different electronic transitions probed. Interband UTA is the most common technique. Here the probe beam is tuned to the lowest energy absorption maximum (LEAM), which corresponds to a transition from a state just below the valance band maximum (VBM) to the conduction band minimum (CBM). (The VBM to CBM transition is typically optically very weak in QDs and thus not well suited to UTA measurements). The absorption of probe photons at the LEAM is dependent on the number of empty states in the CBM, therefore if MEG occurs absorption will decrease and there will be an increase in probe transmittance through the sample. A similar technique is intraband-UTA, where the probe photons are tuned to a transition from the CBM to a higher state; in this case the presence of additional electrons in the CBM results in an absorption increase [9]. Thirdly, THz-UTA probes the frequency-dependent complex conductivity of the sample, from which the exciton populations can be determined through the Drude model of free carriers [10].

#### 2.1.2. Photoluminescence

Photoluminescence (PL) measurements have also been used to determine MEG, and several variants have been developed. One method utilises the time-correlated single-photon counting technique to measure the components of the PL decay transient associated with single and multi-exciton recombination [11]. This method benefits from the time-resolution and sensitivity of the micro-channel plates (MCPs) used, which allows biexciton decay to be resolved and the weak signals resulting from the low excitation rates needed for MEG measurements to be readily detected. However, the spectral range of MCPs limits their use to QDs with band gaps larger than ~1.5 eV, *i.e.*, greater than the ideal value for the exploitation of the solar spectrum. Similarly, MEG has also been measured by detecting the PL decay with a streak camera [12]. The PL signal detected following quasi-continuous wave excitation has also been used, benefiting from the sensitivity of a photo-multiplier tube (PMT) used as a detector [13]. However, this method does not reveal the details of the multi-exciton recombination dynamics and the PMT response limits the range of emission wavelengths that can be detected. Ultrafast PL up-conversion (UPLU) circumvents the spectral limitations of sensitive PMTs by sum-frequency mixing the PL photons with an ultrafast laser pulse in a non-linear crystal [14,15]. The output is thus a blue-shifted signal with an amplitude proportional to the PL intensity. By controlling the laser pulse wavelength, the exact wavelength of the blue-shifted signal can be adjusted to be within the spectral range of the detectors. Moreover, since up-conversion only occurs whilst the laser pulse is co-incident with the PL in the nonlinear crystal, varying the arrival time of the laser pulse enables the PL decay to be resolved down to the laser pulse duration, typically ~0.1 ps. Finally, PL quantum yield measurements have been used to deduce the existence of a novel variant on the MEG process in close-packed arrays of Si QDs [16], as described in Section 3. In this case, the additional excitons are generated not in the same QD that absorbed the photon, but rather in a neighbouring QD. This separation enables the extra excitons to survive long enough to make a significant contribution to PL emission.

#### 2.1.3. Transfer to Molecular Complexes

Implementation of MEG in practical devices requires efficient extraction of MEG charges before their ultrafast recombination. This has led to the study of exciton dissociation by charge transfer to electron- or hole-acceptor molecules bonded to the QD surface. Surface treatment of QDs can however inhibit the MEG process with some studies reporting a strong variation in MEG efficiency [17,18,19]. One group, however, has shown using UTA that in PbS QD/methylene blue complexes the number of dissociated excitons is equal to the number of excitons generated in free QDs under the same excitation conditions, with MEG and multiple exciton dissociation efficiencies of 112% [20].

#### 2.1.4. Interpreting Data

As discussed above, the formation of multi-excitons is possible not only by MEG but also by the absorption of more than one photon by a QD during a pump pulse. Therefore, the observation of multi-exciton decay in transient absorption or PL studies can only be taken as evidence of MEG if the pump fluence can be confirmed to be sufficiently low as to make the chance of a QD absorbing two or more photons per pump pulse negligible. The probability of a QD absorbing *N* photons per pump pulse, *P*(*N*), is determined by Poisson statistics, and depends on the absorption cross section of the QD at the pump wavelength, σ, and the pump fluence, *J* (in units of photons per pulse per unit area):

*P*(*N*) = 〈*N*〉^*N*^ exp(−*N*)/ *N* ! (1)

where the average number of photons absorbed per QD, <*N*> = σ*J*. Thus, in order to calculate whether there is a negligible chance of this alternate multi-exciton formation process occurring, knowledge of σ is required. However, σ is difficult to determine accurately, and a suspected underestimation of σ, and thus of <*N*>, was one explanation suggested for the discrepancy in the early results reported by different groups for nominally similar QDs [21]. (Other explanations suggested at that time included variation in <*N*> due to pump beam inhomegeneities and variation in the surface chemistry between samples [1].)

**Figure 3:**
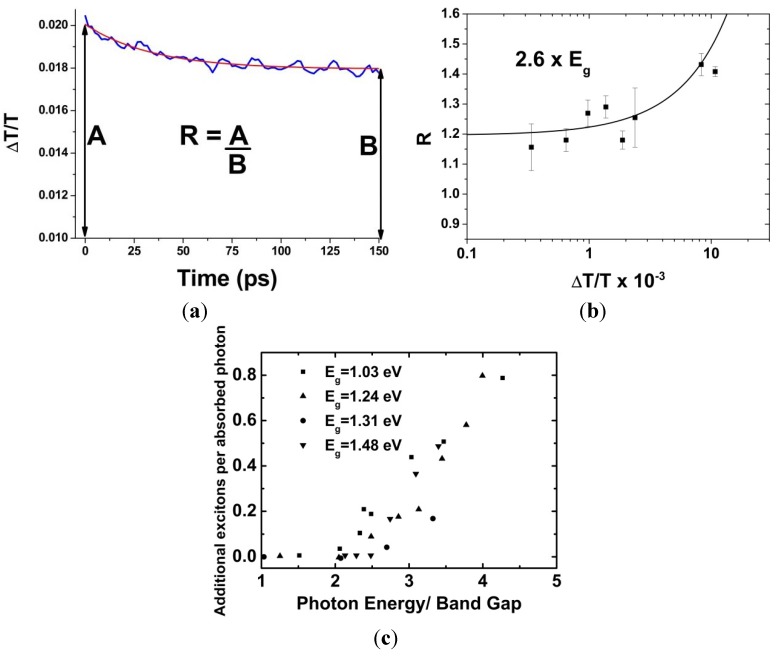
(**a**) A typical signal obtained from a UTA experiment showing the fractional transmittance change, Δ*T*/*T*, as a function of time delay between the pump and probe beams. *R* is the ratio between the peak amplitude, *A*, and the plateau amplitude, *B*, where enough time has passed that all bi-excitons have decayed, but before any significant decay of single-excitons; (**b**) *R* as a function of fractional transmittance change, Δ*T*/*T*, for InP QD excited at 2.6 times *E*_g_. The line is a fit to Equation (2), data from Reference [24]; (**c**) Additional excitons produced, (QY − 1), as a function of *hv*/*E*_g_ for InAs NQD of different *E*_g_. *hv*_th_ corresponds to the point where the rise in additional excitons begins, data from Reference [25]. Reproduced from Reference [26] by permission of the PCCP Owner Societies.

Consequently, an analysis method was developed that did away with the need to know σ. A typical pump-induced transmittance transient is shown in Figure 3a. *R* is the ratio of the signal maximum, which is proportional to the average number of excitons created per QD, and the signal at a time significantly greater than the biexciton lifetime but significantly less than the single exciton lifetime. This latter time corresponds to when all multi-excitons created by the pump pulse have decayed to single excitons, but the resulting single exciton population has largely yet to decay. In the limit of low fluence, each excited QD has absorbed only one photon and so the population of single excitons at this latter time is equivalent to the number of absorbed photons. Thus, in this limit, *R* corresponds to the average number of excitons created per absorbed photon, *i.e.*, the QY. The low fluence limit of *R* can be found experimentally by measuring *R* for a range of pump fluences and fitting the following equation to the resulting data [22]:

*R*(*J*) = *QYσJ*[1 − exp(−*σJ*)]^−1^ (2)

Figure 3b shows an example of this analytical procedure for MEG in InP QDs. In this case, *R* has been plotted against the maximum value of the fractional transmittance change transient, Δ*T*/*T*|_max_, which is proportional to <*N*> [23]. Repeating this process for different pump wavelengths enables the number of additional excitons per absorbed photon, (QY − 1), to be found as a function of photon energy normalized to *E*_g_, as shown in Figure 3c. From this, the values of *hv*_th_ and η can be determined by finding the intercept and gradient of a linear fit to the data for which QY > 1.

When analysing ultrafast PL decay, there is the added complication that the initial maximum intensity depends on both the biexciton recombination rate *k_xx_* and the single exciton recombination rate *k_x_*, whilst at the plateau only on *k_x_*. Analysis of ultrafast PL data thus requires the ratio of these rates to be known. The value of *k_xx_*/*k_x_* initially used by various authors ranged between 2 and 5 [11,12,14] and indeed the discrepancy between these values was suggested as the source of the variation in the reported MEG efficiencies [14]. However, by considering that the multi-exciton recombination rate is equal to *k_x_* multiplied by the number of different ways a multi-exciton’s constituent electrons and holes can recombine with each other, a more recent work has established that for PbSe QD *k_xx_*/*k_x_* = 4 [15].

### 2.2. Artefacts

#### 2.2.1. Trion Recombination

**Figure 4:**
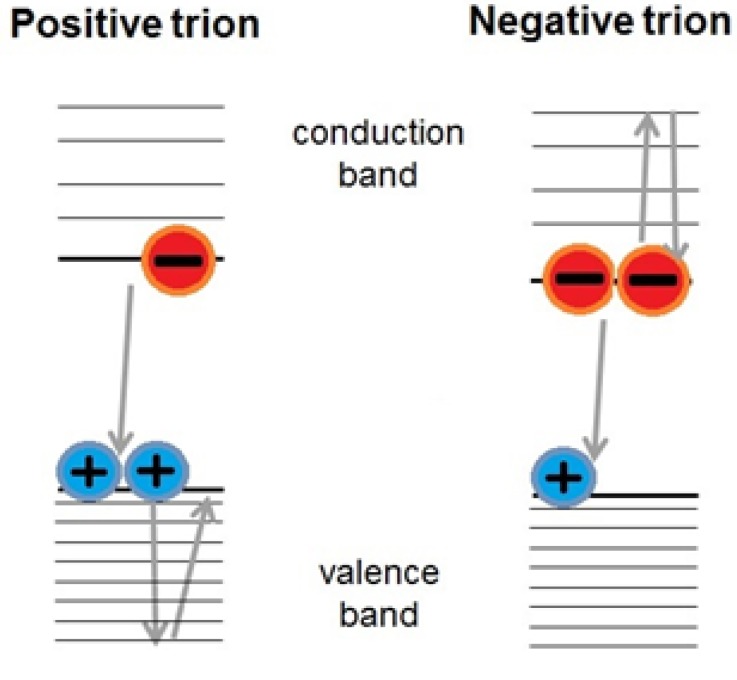
Trion formation in a QD provides a charge capable of receiving and dissipating the energy liberated in the recombination of an exciton, resulting in an enhanced single-exciton recombination rate.

As discussed above, a signature of MEG is, at low pump fluences, a rapid signal decay on the sub-nanosecond time-scale corresponding to fast Auger recombination of multi-excitons, a process inaccessible to single-excitons. However, fast Auger recombination can also occur when a trion is formed within the QD, *i.e.*, an excitation involving three charge carriers—see Figure 4. Trions can form when a hole or electron is trapped on the QD surface for a time longer than the period between excitation pulses. Its geminate charge is thus already present within the QD when an exciton is formed following photon absorption during a subsequent pump pulse. The unpaired electron or hole residing within the QD is able to receive the energy liberated in recombination, enabling fast Auger recombination of the remaining electron–hole pair. This process can produce a sub-nanosecond decay in the signal even at low pump fluences and thus can resemble the signature of MEG. However, several careful [27,28,29] studies have shown that sufficient stirring or flowing of the sample can refresh the QDs within the excitation volume in between pump pulses, preventing the formation of trions and thus of this misleading component to the signal.

#### 2.2.2. Direct Surface-Trapping

Direct surface-trapping is another process which has been shown to produce significant artifacts in UTA experiments that can be mistaken for MEG, including apparent MEG yields of 170% while below *hv*_th_ [30]. The surface of QDs can mediate recombination routes for excited charges and this was shown to enable a rapid sub-nanosecond decay of the CBM electron population that resembles multi-exciton decay at low pump fluence, *i.e.*, it mimics the signature for MEG. It was also demonstrated that the presence and effect of surface states is significantly increased following prolonged exposure to high intensity radiation. However, the presence of these surface states produces a characteristically broad pump-induced absorption feature at energies just below the LEAM, which can be used to distinguish this effect from MEG. Moreover, surface-mediated recombination results in a non-monoexponential form to the decay transients associated with this process, in contrast to the mono-exponential decay due to multi-exciton decay; this provides another way in which the two processes may be distinguished. It has also been demonstrated that QD surface passivation methods, such as surrounding a CdSe core with a ZnS shell [30], can ameliorate surface state formation and hence reduce or eliminate this recombination pathway.

## 3. Dependence on QD Properties

### 3.1. Material Composition

#### 3.1.1. Pb Chalcogenide QDs

The first demonstration of MEG was in PbSe QDs [8] and these have been the subject of more studies than any other QD type. QDs of PbS and PbTe have also been investigated but to a lesser degree [10,14,28,31,32,33,34,35], and there have also been some recent studies of alloyed PbSe*_x_*S_1−*x*_ QDs [34]. The reason for this focus on lead chalcogenide QDs is largely due to the small *E*_g_ of the bulk materials (e.g., 0.28 eV for PbSe [1]), which enables the absorption edge of the corresponding QDs to be size-tuned to the optimum value for exploitation of the solar spectrum. As will be discussed in Section 5, the greatest efficiency is achieved from a conventional solar cell for *E*_g_~1.35 eV but this value is shifted further into the infrared for a cell benefiting from MEG. For all the types of Pb chalcogenide QD, almost all studies report a *hv*_th_ value in the range 2.6–3.0 *E*_g_ that is independent of NC diameter (with the exception of Reference [32] which describes *hv*_th_ reducing with decreasing QD diameter). However, two recent works [34,36] compared η for PbS, PbSe and PbSe*_x_*S_1−*x*_ QDs and found a difference in the size-dependence between PbSe and the other materials. In particular, for PbSe QDs η was approximately constant with QD size for the samples studied at ~0.4, whilst for PbS and alloyed QDs it was found to increase with decreasing diameter, for instance ranging from η~0.26 to η~0.4 as the diameter of a PbS QD reduced from 9.4 to 4.2 nm [34]. The authors were able to reconcile these results by showing that each material exhibited the same linear increase in η with decreasing size when the physical radius of the QD was normalized to the radius at which the confinement energy equals the Coulomb energy [34].

#### 3.1.2. Cd Chalcogenide QDs

The second class of QDs to be the subject of MEG studies, starting in 2005, were those composed of Cd chalcogenides. Cd-based QDs are not well-suited to the exploitation of MEG in photovoltaic applications because they have too large a *E*_g_ to effectively harvest the solar spectrum. However, the synthetic techniques by which they are fabricated are mature and the well-controlled samples that result are useful for exploring the underlying physical processes relevant to MEG. MEG was first reported in CdSe QDs by Schaller *et al*. [37] and had a threshold of 2.5*E*_g_. This was particularly noteworthy because it demonstrated that thresholds lower than ~3*E*_g_, the lowest value that at that time had been measured, could be achieved. As will be discussed in Section 5, a threshold close to the energetic limit of 2*E*_g_ is key to realizing the potential benefit of MEG to solar cell efficiency. Later studies on CdTe [38] and CdTe/CdSe core/shell [13] QDs also reported similar threshold values of ~2.5*E*_g_ and 2.65*E*_g_, respectively. However, in 2007 Nair *et al*. [12] found an absence of MEG in both CdSe and CdTe QDs, and the discrepancy between this and other reports contributed to the realization by the community that MEG efficiency measurements were being confused by experimental artifacts (as described in more detail in Section 2.2). A later theoretical study [39] showed that this discrepancy could plausibly be explained by the effects of photo-induced surface trapping. MEG in CdSe and CdTe QDs has not yet been revisited since the experimental techniques that are now used to suppress these artefacts were developed and so it is not possible to quantify η with confidence in these materials. In contrast, the experimental technique used to monitor MEG in the CdTe/CdSe NCs does allow an upper limit to the efficiency of η~0.5 to be inferred [13].

#### 3.1.3. InAs and InP QDs

Interest in InAs QDs was based both on the low *E*_g_ of the bulk material, which enables size-tuning of the band edge to the optimum for solar cell efficiency, and on the expectation that the MEG threshold would be close to the energetic minimum of 2*E*_g_. This expectation arose from an analysis of how the excess energy of an absorbed photon is divided between the photo-generated electron and hole [37] and yields the following expression:

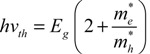
(3)
where *m*_e_^*^ and *m*_h_^*^ are the effective masses of the electron and hole respectively. For InAs, *m*_e_^*^ << *m*_h_^*^ and thus *hv*_th_ ≈ 2*E*_g_. The first study of MEG in InAs QDs appeared in 2006 in a paper by Pijpers *et al*. [40] and reported a quantum yield of 160% for a photon energy of 2.7*E*_g_ but did not measure *hv*_th_ or η. A study by another group in the following year [25] studied how the MEG quantum yield varied with the energy of the absorbed photons in InAs QDs and hence could determine these quantities, reporting *hv*_th_ ≈ 2*E*_g_, as expected, and also η~0.35. However, Pijpers *et al*. [41] later retracted their results after failing to reproduce them. A subsequent study by some of the authors [21], which included new experimental procedures designed to remove the need to determine the wavelength-dependent absorption cross-section of the NCs, also concluded that MEG did not occur in their samples up to a photon energy equivalent to 3.7*E*_g_. In 2009, modeling by Califano [42] found that trion and biexciton decay lifetimes, though similar, should be experimentally distinguishable in InAs NCs, suggesting that some other process was responsible for the discrepancies in reported MEG quantum yields. Very recently, a combined experimental and theoretical study [43] also found that the MEG quantum yield was negligible for photon energies up to at least 3.2*E*_g_ for InAs QDs with a *E*_g_ close to optimum value for solar cell efficiency. At low pump fluences, there was no difference found between the transients obtained from static and well-stirred samples, and hence the sub-nanosecond decays observed were attributed to surface-trapping. Furthermore, the results of the theoretical modeling found that, while MEG is energetically possible for photon energies similar to *E*_g_, the efficiency of MEG only becomes significant close to *hv*_th_ for small QDs, the *E*_g_ of which is too large to effectively exploit the solar spectrum.

The large *E*_g_ values of InP QDs (~1.7–2.0 eV) [44] makes them unsuited to the enhancement of solar cell output by MEG. However, as for InAs, *m*_e_^*^ << *m*_h_^*^ for bulk InP and so the minimum value of *hv*_th_ was also expected for MEG in InP QDs. Considering the controversy surrounding the measurement on InAs QDs, an investigation of MEG in InP QDs was important in order to confirm that *hv*_th_ ≈ 2*E*_g_ was indeed achievable, and thus that high *hv*_th_ values observed for the Pb- and Cd-chalogenides were not universal and the full benefit of MEG to solar cell efficiency could, in principle, be realized. The first, and to date only, investigation of MEG in InP QDs was undertaken in 2009 [24], at a time when the causes of the experimental artifacts that had confused earlier measurements had become apparent. Consequently, measurements were taken using well-stirred samples and at moderate pump fluences. The InP core of these QDs was surrounded by a ZnS inner shell and a ZnO outer shell; these layers of high *E*_g_ materials were designed to prevent, or at least reduce, the trapping of photo-generated charges. These precautions enabled values of *hv*_th_ = 2.1*E*_g_ and η = 0.3 to be reported with confidence; this threshold is consistent with Equation (3) and remains the lowest reported value since the causes behind the experimental artifacts affecting MEG measurements were recognized.

#### 3.1.4. Silicon QDs

Silicon is an abundant, low-toxicity material and its semiconductor properties have been very well studied, all of which make it a strong candidate for MEG-enhanced solar cells. The first demonstration [22] of MEG in Si utilized colloidal QDs with a diameter of 9.5 nm and *E*_g_ = 1.2 eV, and reported *hv*_th_ = 2.4 *E*_g_ and a quantum yield of 2.6 for a pump photon energy of 3.4 *E*_g_, which corresponds to η = 1.6. Note, however, that this study was undertaken in 2007 before the experimental artifacts that can affect MEG measurements were fully understood.

Recent investigations of MEG in close-packed Si QDs embedded in a silica matrix have yielded some interesting and potential important results. First by using photoluminescence quantum yield measurements [16] and then subsequently ultrafast transient absorption spectroscopy [45], a group based at the University of Amsterdam has reported the step-like enhancement in the quantum yield of photo-generated charges that has been predicted for very high efficiency MEG. Intriguingly, their experiments also indicate that the additional excitons are being generated not in the same QD that absorbs the photon, as is the case for the essentially isolated QDs found in colloidal samples, but rather in a neighboring QD. This is an exciting result not only because it suggests that the efficiency of MEG in real systems can approach the ideal case, and hence the full benefit to solar cell efficiency could potentially be realized, but also because it indicates that some hitherto unforeseen aspect of MEG is at work. However, as yet, these results await confirmation by other groups and so it is too early to judge their full impact.

#### 3.1.5. Semi-Metal QDs

A theoretical analysis of the relative performance of various QDs led Allan and Delerue to propose in 2011 [46] that the greatest MEG efficiency could be expected for the materials with the smallest bulk *E*_g_. In effect, this corresponds to maximizing the confinement of the carriers whilst still achieving an absorption edge optimized for the solar spectrum. The materials with the smallest bulk *E*_g_ are semi-metals, *i.e.*, materials with a zero or small negative value of *E*_g_, and Allan and Delerue took the case of α-Sn QDs as an example in their study. However, the first experimental study of MEG in a semi-metal used instead QDs made from HgTe, which has a bulk *E*_g_ = −0.15 eV [23]. For 3.5 nm QDs with *E*_g_ = 1.0 eV, a quantum yield of 1.36 was measured for a photon energy equivalent to 2.9*E*_g_; this compares well with Pb-chalogenide QDs which typically exhibit a quantum yield of ~1.2 for excitation at ~3.0*E*_g_. HgTe QDs are also notable for their resistance to oxidation—in this case, the QDs were prepared in water and open to the ambient atmosphere. In contrast, Pb-chalocogenide QDs are susceptible to significant oxidation unless kept under an inert atmosphere [28,47].

### 3.2. Structure

#### 3.2.1. Core-Shell Nanocrystals

Several of the QDs studied to date have had a core-shell structure, where a nominally spherical QD of one material is surrounded by one or more layers of different semiconductor material. For instance, as mentioned in Section 3.1.3, the InP NCs used to demonstrate the record low MEG threshold consisted of an InP core surrounded by a ZnS inner shell and a ZnO outer shell. Similarly, the InAs QDs studied have had cores over-coated by layers of CdSe [25], ZnSe [43], or CdSe and ZnSe [21]. In these cases, the purpose of the shell layer(s) is the same: to act as a barrier between both photo-generated charges (electron and hole) and the QD surface, and thereby reduce or eliminate surface-mediated relaxation and recombination.

A core-shell QD is described as having a Type I heterostructure if both carriers are confined to the same volume, such as in the InP and InAs QDs described above. In contrast, a core-shell QD in which the electron localizes in the core and the hole in the shell, or *vice versa*, is known as a Type-II heterostructure. The intermediate case, in which one of the carriers is localized to either the core or the shell and the other de-localised over the whole QD, is called a quasi-Type II heterostructure. The rate of Coulombic interactions between the electron and hole, including Auger cooling, depends on the overlap of the wavefunctions of the two carriers and this is reduced in Type II and quasi-Type II heterostructures. As will be described in more detail in Section 6, Auger cooling of hot electrons is one of several processes that compete with MEG, and so its suppression may lead to an improved quantum yield. Gachet *et al*. investigated MEG in CdTe/CdSe/CdS/ZnS QDs, in which the inner CdTe/CdSe core/shell forms a Type-II heterostructure and the outer CdS and ZnS shells form a barrier between the photo-generated charges and the QD surface [13]. A value of *hv*_th_ ≈ 2.65*E*_g_ was observed, consistent with other studies of Cd-based QDs (see Section 3.1.2), and an upper limit of η = 0.53 estimated, a value second only to that reported for Si QDs (since the identification of the misleading artefacts described in Section 2). Quasi-Type II PbSe/PbS and PbSe/PbSe*_x_*S_1−*x*_ QDs have also been investigated [36] but in that case no improvement in MEG efficiency was noted, which was attributed to the small reduction in wavefunction overlap calculated for these QDs.

#### 3.2.2. Nanorods

There have been several investigations of MEG in PbSe nanorods, *i.e.*, NCs elongated in one direction to, typically, a few ten seconds of nanometers. These studies were motivated by the expectation that MEG would be enhanced in these structures, both by an enhancement of the Coulomb interactions between carriers due to the quasi-one-dimensional confinement [48], and also by an increased density of multi-exciton states due to the reduced symmetry of the nanorod compared to a QD [49]. One group found that MEG measurement on nanorods with an aspect ratio (*i.e.*, length/diameter) between 5 and 8 yielded *hv*_th_ = 2.6*E*_g_ and η = 0.37 [49], similar to the results for PbSe QDs reported by the same group that year [34]. This was somewhat of an improvement on the results of *hv*_th_ = 2.7*E*_g_ and η = 0.25 for PbSe QDs measured in the same laboratory previously [15,27], but was within the range of values reported elsewhere for PbSe quantum dots—see Section 3.1.1. However, a previous study by another group [50] reported on similar PbSe nanorods and found, after a subsequent correction [51], *hv*_th_~2.5*E*_g_ and η~0.5 (note that for comparability with the other values given in this review the *hv*_th_ value given here is estimated by linear extrapolation of the data to when the quantum yield reduces to unity, rather than by a fit to the phenomenological model described in Section 4.1 as performed by the authors of Reference [51].)

### 3.3. Surface

The primary purposes for seeking to control the surface of QDs is to ameliorate the processes discussed in Section 2 that inhibit accurate measurement of QY and compete with MEG, and to prevent the QD reacting with external agents, potentially altering its properties. Sykora *et al*. [47] investigated the absorption spectra and QY measurements of PbSe QDs exposure to air for varying times. They found that the QDs can lose up to 50% of their volume due to oxidation in air, resulting in smaller QD cores. This enhances quantum confinement and blue-shifts the absorption peak, moving it further from the optimum value for exploitation of the solar spectrum. However, they also showed that the effects of air exposure are partially suppressed in PbSe/CdSe core/shell structures, similar to the CdSe/ZnS structures used by Tyagi *et al*. [30] to ameliorate surface state formation. Similar effects of air exposure have also been reported for PbS QDs [28,47].

Typical surface passivation methods result in long-chain ligands bonded to the QD surface. In thin-film QD solids these long-chain ligands are replaced with shorter ones to enhance carrier mobility; the shorter inter-QD distances enhance carrier tunneling between QDs [52,53,54,55]. This process was observed to reduce MEG efficiency, but this reduction could be reversed through infilling with inorganic compounds [56,57,58]. The authors investigated MEG in 1,2-ethanedithiol-linked PbSe QDs infilled with Al_2_O_3_ or Al_2_O_3_/ZnO through atomic layer deposition. Through time-resolved microwave conductivity measurements they found MEG efficiencies for infilled films approached the intrinsic efficiency for isolated PbSe QDs, whereas there was negligible MEG for non-infilled films.

## 4. Understanding MEG

### 4.1. Phenomenology

Beard *et al*. [59] have shown that the overall efficiency for the MEG process can be related phenomenologically to the threshold energy by:

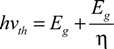
(4)


This relationship implies that reducing the threshold energy of the MEG process also increases η. In addition Beard *et al*. [59] (following the work of Ridley [60]) parameterised the competition between the possible relaxation routes of a single hot exciton by:

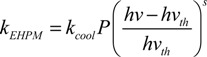
(5)
where *k*_EHPM_ is the rate of cooling by electron hole pair multiplication (EHPM), *i.e.*, MEG, *k*_cool_ is the rate of cooling by energy dissipation, the exponent *s* can vary between 2 and 5, and *P* is the threshold parameter which determines whether the MEG onset is hard (*P* >> 1) or soft (*P* < 1). An efficiency can then be defined based on *P*:

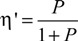
(6)
which allows the parameterisation of an energy threshold for each additional EHPM event:

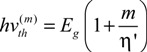
(7)
where *m* is the maximum number of EHPM events possible. Through Equations (5)–(7) the onset of MEG and the efficiency can be modeled for varying levels of competition between EHPM and carrier cooling. It was shown that the onset of MEG is not as sharp as expected, and that the QY increases approximately quadratically below 150%, and linearly above.

### 4.2. Comparison to Impact Ionization in Bulk Materials

Interest in MEG in QDs as a source of increased efficiency in photovoltaics was fuelled by the expected effects of quantum confinement, namely relaxed momentum conservation, slower phonon-mediated cooling rates, enhanced Auger processes and reduced dielectric screening of the QD surface [10,59]. During the period of controversy following the emergence of the artefacts described in Section 2, several groups argued that for a given photon energy, carrier multiplication occurs more efficiently in bulk PbS and PbSe than in QDs of the same material [10,61]. It was suggested that surface effects in competition with impact ionization are much less prominent in bulk materials due to a greatly reduced surface area to volume ratio compared to a QD. Conversely, it was also argued that the MEG threshold in bulk materials is much higher due to parabolic energy momentum states demanding strict momentum conservation in exciton production.

In order to effectively compare the performance of bulk materials and QDs, careful consideration of the data presentation is required. As discussed by McGuire *et al*. [15] arguments can be made for superior performance of either depending on the quantities displayed; when plotting the QY *versus* the pump photon energy, bulk PbSe was shown to outperform PbSe QDs and this was the representation used by authors for the notion that CM in QDs is less efficient than in bulk [10,14]. This representation, however, neglects the effect of the magnitude of the band gap and does not provide direct information about the energy efficiency, only the number of excitons produced per absorbed photon. Quantum confinement in QDs results in significantly higher band gaps meaning an MEG event corresponds to higher final energy state than an impact ionization event in bulk material. A representation which takes these factors into account is to plot the energy yield, QY × *E*_g_, *versus* the photon energy. In this way PbSe QDs were shown to transfer more energy to charge carriers than bulk material for a given photon energy, the more important consideration for photovoltaics.

### 4.3. Theoretical Models

**Figure 5:**
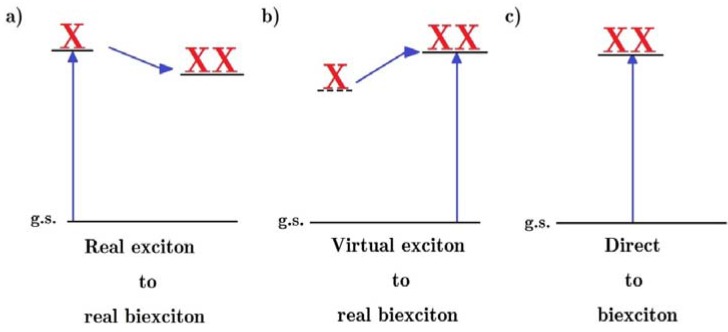
Models of biexciton formation from a single absorbed photon: (**a**) a photo-generated hot exciton relaxes by impact ionization, exciting another electron across the band gap; thereby creating a biexciton (**b**) the direct photogeneration of a biexciton through a virtual exciton or biexciton states [62]; and (**c**) the photo-generation, by a single photon, of a quantum superposition of all possible excited states, including multi-excitons.

Several different theoretical descriptions of MEG have been studied: impact ionization, in which the hot exciton initially created by the absorption of a high energy photon gives its excess energy to one or more valance band electrons so that they are promoted across the band gap; the generation of a biexciton via a virtual exciton or biexciton; and the initial photo-excitation of a superposition of quantum states corresponding to all possible excited states, including multi-excitons. These different models are illustrated in Figure 5 for the simplest case of MEG, where just one additional exciton is created.

One of the earliest experimental studies of MEG [62] also first suggested that the direct photogeneration of biexcitons through a virtual electron channel could also contribute to the MEG quantum yield, in addition to the contribution from impact ionization. A subsequent work suggested that MEG could also proceed via a biexciton channel, further enhancing the yield and argued, based on estimates of the efficiency of MEG via these channels, that this was, in fact, the dominate process [63]. However, a more recent study incorporated selection rules and oscillator strengths into the same model, and concluded that that the efficiency of these channels was much weaker than previously estimated [64]. Moreover, another recent investigation has demonstrated that the lack of interference between photo-generation pathways due to the fast de-phasing brought about by elastic interactions with the crystal lattice further reduced the contribution of this effect to overall MEG yield [65].

The direct photo-generation of multi-exciton states as part of a coherent superposition of all possible excited states was first proposed in 2006 [66] as another process which contributes to the overall MEG yield. In this model, this direct photo-generation is made possible by the strong many-body interactions produced by quantum confinement. However, the rapid de-phasing of the constituent single and multi-exciton states within 10 fs is again brought about by interactions with the lattice; the multi-exciton states then subsequently, within 100 fs, fission into multiple uncorrelated single excitons [67]. This process is thought to be greatest for QDs with strongly correlated electrons, *i.e.*, for QD radii much smaller than the exciton Bohr radius, and low dielectric constants, which reduces the screening of the inter-particle interactions [67]. However, recent studies which introduced these de-phasing effects into the original model concluded that this process does not make a significant contribution to overall yield for the PbSe QDs modeled and that impact ionization is the dominant process [65,68].

MEG in QDs by impact ionization has been studied by a number of groups, using several approaches to the calculations. The impact ionization rate, *W*, is calculated using Fermi’s golden rule:

*W* = 2π│*V_if_*│^2^ ρ_*f*_(*E_i_*)/*h* (8)

where ρ_f_(*E*_i_) is the density of final states and *V*_if_ is the screened Coulomb interaction matrix element. Atomistic pseudo-potential calculations have been performed for PbSe QDs [69] but yielded *hv*_th_ = 2.2*E*_g_, somewhat lower than the experimentally observed value. Screened semi-empirical pseudo-potential calculations of *W* in CdSe and InAs QDs [70] found a decrease in MEG efficiency with increasing QD size, with the process becoming inefficient for diameters greater than 3 nm. Allan and Delerue have used a semi-empirical tight-binding model to calculate *W* in a range of QD types, including PbSe [10,61,71], PbS [10], InAs [43], Si [72], α-Sn [32], and HgTe [23]. In particular, it was found that while *V*_if_ increases as the QD size is reduced, due to increased Coulomb interactions between particles, ρ_f_(*E*_i_) is decreased to an even greater degree such that *W* is reduced overall. It was also found that good agreement could be found between the calculated and experimentally measured values of *hv*_th_ and η if the rate of carrier cooling by the processes in competition with impact ionization was given a value of a few picoseconds, consistent with measurements [23,61,71].

In summary, calculations can reproduce experimental MEG results by considering only the rate of impact ionization relative to the rate of carrier cooling, the other process that could potentially contribute to MEG yield being made inefficient by the de-phasing of the corresponding coherent states due to interactions with the QD crystal lattice.

## 5. Device

### 5.1. Potential Benefit to Solar Cell Efficiency

**Figure 6:**
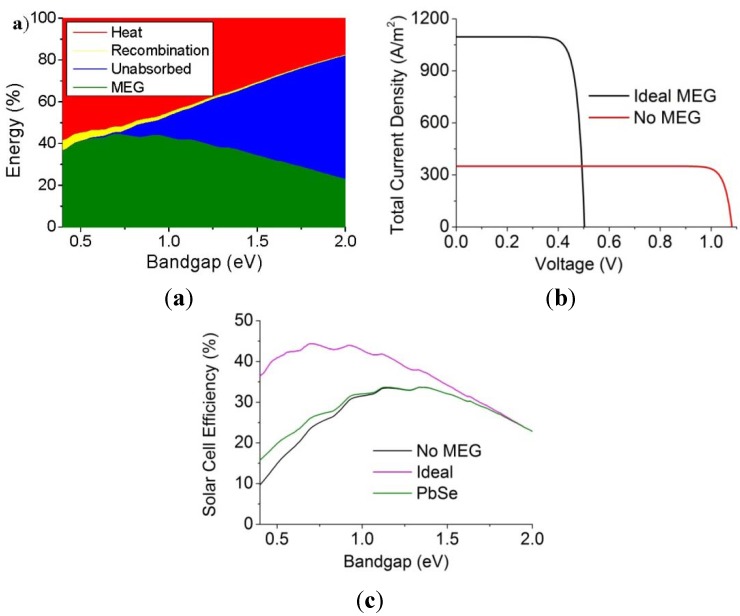
(**a**) Energy division in a QD sample exhibiting ideal MEG modeled using Equation (12) if illuminated by the AM1.5 solar spectrum. As the bandgap increases a larger fraction of solar photons do not have enough energy to be absorbed, indicated by the blue section, and for lower bandgap the threshold for MEG is lower; (**b**) Comparison of current density as a function of operating voltage for QD solar cells of optimum *E*_g_ with and without ideal MEG; (**c**) Comparison of solar cell efficiency *vs.* *E*_g_ simulated using Equation (13), for PbSe QD with experimentally derived η and *hv*_th_, to the ideal case from (**a**) and the case with no MEG.

Theoretical efficiency limits on conventional solar cells have been explored using the “Detailed Balance” model [2] and modifications have been made to model the potential benefits of MEG [73,74,75]. In the ideal case, it is assumed that all photons with an energy greater than *E*_g_ are absorbed and produce a number of excitons dependent on *hv* and *E*_g_, described by QY (*hv*,*E*_g_). The photogenerated current density, *j*_pg_, is thus given by:

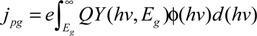
(9)
where e is the electronic charge and ϕ(*hv*) is the spectral photon flux density. This is offset by a loss of photogenerated current due to recombination. The recombination current density is given by:

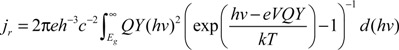
(10)
where *h* is Planck’s constant, *c* is the speed of light, *V* is the operating voltage of the cell, *k* is Boltzmann’s constant and *T* is the temperature (set to 300 K here). *V* is assumed equal to a constant quasi-Fermi level separation and is determined by finding the value which maximizes the photo-voltaic efficiency of the cell:

η_pv_ = *jV* / *I* (11)

where *j* = *j*_pg_ − *j*_r_, the total current density, and *I* is the total solar irradiance (here set to AM1.5). An assumption must be made on the exact form of QY (*hv*,*E*_g_). For optimized MEG a photon produces an additional exciton for each increase in energy of *E*_g_, thus:

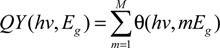
(12)
where θ (*hv*,*mE*_g_) is the Heaviside step function (equal to 1 for *hv* > *mE*_g_, 0 otherwise), and *M* is the integer found from rounding down *hv*_max_/*E*_g_. Note that limiting *m* to 1, limiting QY to 1, *i.e.*, no MEG. Figure 6a illustrates how, for the case of ideal MEG and for a range of *E*_g_ values, the incident solar energy is divided between the processes of MEG, radiative recombination and waste heat generation, with the balance being accounted for by photons with a wavelength too long to be absorbed. At the optimum *E*_g_ value of 0.7 eV, the cell efficiency is 44%; in comparison, without MEG the optimum efficiency is 33%, and occurs at *E*_g_ = 1.35 eV. Figure 6b compares the current density-voltage characteristics of a cell benefiting from ideal MEG to one for which MEG is negligible: MEG increases the short-circuit current density by a factor of ~3 whilst decreasing the open-circuit voltage by a factor of ~2.

Alternatively, a linear increase in QY above *hv*_th_, which better corresponds to the experimental observations to date, can be described by:

*QY*(*hv*,*E_g_*)=θ(*hv*,*E_g_*)+ηθ(*hv*,*hv_th_*)(*hv*−*hv_th_*)/ *E_g_* (13)

Using Equation (13), Figure 6c shows how the photovoltaic efficiency varies with *E*_g_ for PbSe NQD with experimentally determined η and *hv*_th_ values of 0.4*E*_g_ and 2.6*E*_g_ eV respectively, as described in Section 3. The AM1.5 solar spectrum was used with a total irradiance of 1 kW·m^−2^. Also included for comparison are the efficiencies for ideal MEG from Figure 6a, and no MEG. It is evident that only a marginal increase in cell efficiency is attainable at low *E*_g_ using PbSe QD. In order to realize significantly enhanced solar cell efficiency, QDs with *E*_g_ = 0.7–1.3 eV and a MEG threshold close to the minimum required by energy conservation, 2*E*_g_, are required.

### 5.2. Real Devices

Due to the ultrashort lifetimes of bi-excitons (~10–100 ps), implementation of MEG in a real photovoltaic device requires charge extraction on a similar time-scale or faster. The first indication that this was achievable was published in 2010 [76], where it was shown that transfer from PbSe QDs to a TiO_2_ surface occurred in less than 50 fs, (and hole transfer in 4 ps to a redox species in solution returning the QD to its ground state). This suggested that device architectures involving QDs adsorbed onto TiO_2_ would be well-suited to the exploitation of MEG.

**Figure 7:**
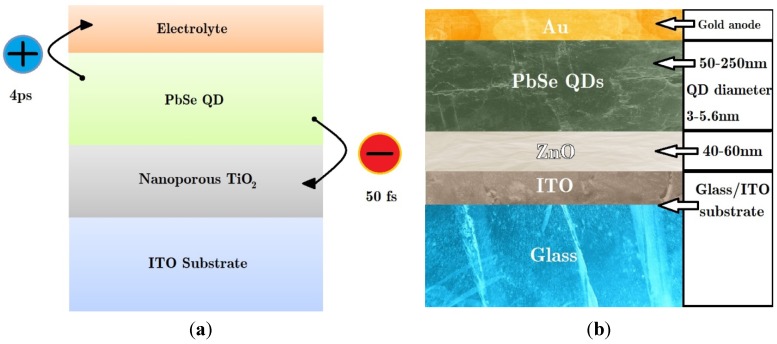
(**a**) Layer structure of a Grätzel-type photovoltaic device utilising PbSe QDs as the absorbing species, with charge carrier transfer illustrated [77]; (**b**) Layer structure of a depleted heterojunction design photovoltaic device utilising PbSe QDs as the absorbing species [78].

The first measured photocurrent enhanced by MEG was reported shortly afterwards from a Grätzel-type photovoltaic device, see Figure 7a. Sambur *et al*. [77] deposited monolayers of mercaptoproprionic-coated PbS QDs of varying band gaps onto an atomically flat TiO_2_ surface, covered by a sulphide-based electrolyte. The cells were illuminated with different wavelengths of monochromatic light; MEG was confirmed by an absorbed photon to electron current efficiency (on a glass/ITO substrate) greater than 100% for photon energies greater than *hv*_th_. The power conversion efficiency was very poor, however, due to low light absorption by the QD monolayer, and so this device did not correspond to a practical solar cell design.

In 2011 demonstration of MEG in a realistic device was demonstrated by Semonin *et al*. [78] in a depleted heterojunction design photovoltaic device utilising PbSe QD as the absorbing species. Such a cell is similar to the Grätzel-type photovoltaic device but with the electrolyte replaced by additional layers of QDs. This design thus benefits from the replacement of a corrosive liquid component, which is prone to leaking, with a thick absorbing layer that also acts as the hole transfer medium. This thick layer of QDs was deposited onto a layer of ZnO deposited onto on a glass/ITO substrate, which acts as a transparent front electrode, see Figure 7c. For photon energies above 3*E*_g_, the external quantum efficiency rose to 114% (corresponding to an internal quantum efficiency of 130%) with an overall power conversion efficiency of 4.5% under AM1.5 simulated sunlight, similar to the best efficiencies for QD-based devices at the time. This indicated that functional photovoltaic devices could benefit from a photocurrent enhanced by MEG under the right conditions.

Future developments in functional devices will require careful examination of the MEG process for QDs within thin films to ensure that the quantum confinement effects which enhance MEG are not compromised in favour of charge mobility. Very recent work by Sandeep *et al*. [58] using time resolved microwave conductance, has investigated the variation of “multiple free charge carrier generation” (MFCG) as the charge mobility is varied by controlling ligand length in PbSe QD films. They report for carrier mobilities in excess of 1 cm^2^ V^−1^·s^−1^, all electrons and holes separate rapidly enough to escape Auger recombination and the MFCG efficiency saturates at a level that is very similar to the CM efficiency for colloidal QD dispersions, with a threshold of 2*E*_g_. This low threshold is the ideal level for efficient MEG, compared to 2.8*E*_g_ for PbSe QDs in solution. The cause of the threshold reduction is not clear and suggests that the relationships derived by Beard *et al*. [59] (Equation (4)) has limitations. Nevertheless, these results are promising for the development of an efficient PV cell enhanced by MEG.

## 6. Future Directions

**Figure 8:**
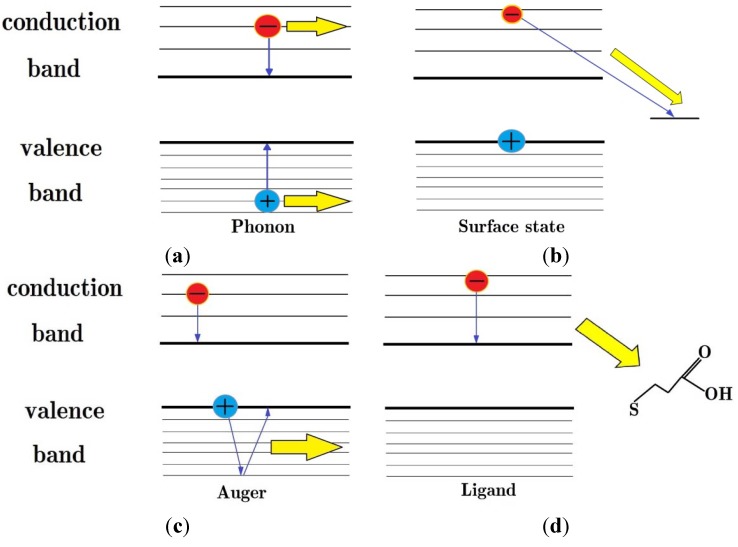
Illustration of the processes which compete with MEG, yellow arrows represent waste energy. (**a**) Phonon emission; (**b**) Electron transfer to a surface state; (**c**) Auger relaxation, where the electron’s excess energy is transferred to the hole which exhibits faster phonon cooling due to the denser band structure of the valence band; (**d**) Transfer of energy to surface ligands through vibrational coupling.

Although current investigations have only reported marginal improvements in energy efficiency for QD-based solar cells due to MEG, there is still significant potential for improvement. One strategy for future development is to utilize the scope QDs afford for control of charge carrier interactions and dynamics [26,33]. This approach can be used to both optimize the absorption edge of the QDs for exploitation of the solar spectrum, and to suppress the processes that compete with MEG. The competing processes include: phonon emission [33], charge carrier transfer to a surface state [43,79], Auger relaxation [80], where the electron’s excess energy is transferred to the hole which exhibits faster phonon cooling due to the denser band structure of the valence band, and transfer of energy to surface ligands through vibrational coupling [81]—these processes are illustrated in Figure 8.

As mentioned in Section 3, core-shell QDs can be produced which enable the localization of the wavefunctions of both the photogenerated hole and electron to be manipulated. This can result in an effective *E*_g_ that is smaller than either of the core or shell material individually, allowing the optimum value to be achieved for a greater range of materials. Manipulating the localization of the charge carriers can also be used to suppress some of the processes in competition with MEG, thereby improving the QY. Reducing the electron-hole wavefunction overlap by the use of a Type II or quasi-Type II QD structure has been shown to dramatically reduce Auger relaxation [81]. Confinement of the electron and hole to the core in type I core/shell QDs can reduce interaction with surface states and ligands [13]. Conversely, QDs which localize charge in the shell may benefit from a more direct charge extraction route, potentially leading to greater extraction efficiency and a consequently improved overall cell efficiency. All of these factors must be considered together in the design of core-shell QD structures.

An additional potential benefit of core-shell QDs is their effect on the bi-exciton interaction energy, Δ*_xx_*. In a Type II or quasi-Type II QD, the imbalance of charge between the core and shell produces a strong Coulombic interaction between the photo-generated charges, the net result of which may be repulsive or attractive. A significant attractive interaction, corresponding to a bound biexciton *i.e.*, Δ*_xx_* < 0, reduces *hv*_th_ by Δ*_xx_* [25]. The Detailed Balance model has been used to calculate the potential to solar cell efficiency of this reduction in *hv*_th_; for Δ*_xx_* = −0.1 eV, the maximum photovoltaic efficiency increases to as much as 50% [82]. However, current experimental investigations have so far found Δ*_xx_* to be repulsive in Type II QDs [83], *i.e.*, Δ*_xx_* > 0. These experimental results are consistent with simple perturbative calculations of Δ*_xx_* [84], which consider just the interactions between the four charges that comprise a bi-exciton as well as self-polarisation effects. However, this approach does not incorporate some important physical effects, such as the exchange interaction [85], and thus does not fully describe the values of Δ*_xx_* achievable. Moreover, other calculations, for ZnSe/CdS QDs with different core diameters and shell thickness in this case, have yielded both positive and negative values of Δ*_xx_* [86,87]. More detailed theoretical studies, including effects such as band bending and electron correlation, are needed to determine whether it is possible to design QDs which exhibit a large and attractive biexciton interaction energy, *i.e.*, Δ*_xx_* < 0, which in an idealized system could result in a threshold energy less than 2 *E*_g_, significantly enhancing MEG efficiency.

## 7. Conclusions

Following the controversy surrounding the initial investigations of MEG, experimental techniques have been developed over the last five years which eliminate misleading artifacts and thus allow the reliable measurement of MEG efficiency. However, while these measurements show that MEG in QDs does result in improved energy conversion compared to impact ionization in bulk semiconductors, the improvement is not currently enough to increase solar cell performance significantly. However, further improvement in MEG efficiency may be realized by engineering exciton dynamics in QDs by control of their size, shape and composition. In particular, suppressing the hot exciton relaxation channels that compete with MEG will significantly improve its efficiency.
